# Influences of Social–Psychological Constructs in Predicting Taiwanese Pro-Environmental Behaviors

**DOI:** 10.3390/bs14040261

**Published:** 2024-03-22

**Authors:** Shin-Cheng Yeh, Alex Yong Kwang Tan, Rei-Ling Lai, Rey-Sheng Her, Wei-Ta Fang, Shiang-Yao Liu

**Affiliations:** 1 Graduate Institute of Sustainability Management and Environmental Education, National Taiwan Normal University, Taipei 116, Taiwan; scyeh@ntnu.edu.tw (S.-C.Y.); wtfang@ntnu.edu.tw (W.-T.F.); 2 Master Program in Sustainability and Disaster Management, Tzu Chi University, Hualien 970, Taiwan; 3 Department of Public Administration, National Cheng Chi University, Taipei 116, Taiwan; reilinglai@gmail.com; 4 Tzu Chi Foundation, Hualien 970, Taiwan; reyrsh@gmail.com; 5 Graduate Institute of Science Education, National Taiwan Normal University, Taipei 116, Taiwan; liusy@ntnu.edu.tw

**Keywords:** pro-environmental behaviors (PEB), value–belief–norm (VBN) theory, Taiwanese, social norms, structural equation modelling (SEM), awareness of consequences, personal values, personal norms

## Abstract

A value–belief–norm (VBN) model for understanding the pro-environmental behaviors (PEB) of Taiwanese was developed. This formulated VBN model included personal values, openness to change, awareness of consequences, personal norms, social norms, and PEB. Ecological world view and ascription of responsibility were excluded to develop a tighter model. A total of 1079 completed questionnaires were collected and structural equation modelling was utilized, where standard estimates and coefficients of determination validated the formulated VBN model’s effectiveness. Each construct served its role as the mediator between its distal variable and outcome variable, with a substantial level of predictive accuracy, and 74.3% of the variance in PEB was accounted for. Further findings found that mature individuals had a stronger tendency towards awareness of consequences due to personal values; the young had a stronger tendency towards personal norms due to awareness of consequences; men had a stronger tendency towards personal norms due to awareness of consequences; and women had a weaker tendency due to a greater emphasis on altruism. Future interventions, such as sharing of personal pro-environmental lifestyles verbally or through social media, periodically decluttering personal items and maintaining a minimalist lifestyle, where these personal norms are in line with collective social norms, could help to strengthen PEB.

## 1. Introduction

The United Nations Intergovernmental Panel on Climate Change released its Sixth Assessment Report (Synthesis Report) in March 2023, stating that during the past ten years, global warming had caused the global temperature of the earth to increase by 1.1 °C above the average temperature of years between 1850 to 1900, creating a range of climate disasters and increasing the pace of climate change. This has led to a loss of biodiversity and has strained the performance of global food, water, and energy systems. In response, the United Nations has repeatedly call for actions to limit the global temperature increases on earth to 1.5 °C above the average temperature of the years between 1850 to 1900, to halve global greenhouse gas emissions by 2030, and to reduce emissions to zero by 2050 [1].

### 1.1. Pro-Environmental Behaviors of Taiwanese

During Earth Day on 22 April 2021, Taiwan officially announced its proposed roadmap and goal to achieve net-zero carbon emissions by 2050 [2]. The Taiwanese government aims to reach net-zero carbon emissions by creating and improving the basic environment for technology research and development as well as through climate legislation. Transformative strategies for transition were planned in the energy and industrial sectors as well as involving lifestyle and social changes for Taiwanese. Lifestyle changes for Taiwanese included the transition of their diet, clothing, housing, and transportation into zero-waste and low-carbon pro-environmental behaviors (PEB). At the same time, re-energizing the social support systems in order to improve mechanisms for resolving conflicts and disputes arising from the transition, could allow the social transition to turn possible conflicts into potential opportunities [3].

Over the years, numerous studies have been conducted to understand the PEB of Taiwanese. Lin [4] examined the PEB of residents from Kaohsiung, Taiwan and their impact on personal diet, clothing, housing, transport, and recreation behaviors. Using the theory of planned behavior, results showed that attitude and perceived behavioral control played a significant role in the research model by positively influencing behavioral intentions. Behavioral intentions significantly influenced PEB only in the category of clothing. It was further pointed out that cultural differences could play an important role and results might be only relevant for Asia countries. Chen [5] studied the PEB of Taiwanese using the Value-Belief-Norm (VBN) theory, explaining 31% of the variances of PEB and noting that the new ecological paradigm, awareness of consequences and ascription of responsibility had a partial mediating relationship between their antecedents and outcome variables. In addition, as people’s perceptions changed over time, follow up studies might be required for longitudinal studies. Yu et al. [6] investigated the purchase intentions and loyalty of Taiwanese undergraduates toward green products. Results highlighted the mediating role of self-responsibility as well as the need to shorten the social distance of green products through outreach of mass media or recommendation from opinion leaders.

In recent years, Tsai and Tan [7] expanded the theory of planned behaviors to include ethical leadership aiming to understanding the PEB of healthcare personals in Taiwan. Ethical leadership was determined to positively influenced attitude, subjective norms and perceived behavioral control. Behavioral intention was found to be not significantly influenced by subjective norms due to the perceived weak social interactions in hospitals during the COVID-19 crisis. 19% of the variances of PEB were explained by this model. Tsai and Tan [8] incorporated moral norms into the theory of planned behaviors to investigate the PEB of undergraduates from a university in Eastern Taiwan. Moral norms was found to positively influenced attitude, subjective norms and perceived behavioral control. PEB were moderately influenced by behavioral intention with 30% of the variances explained by this model. Chang et al. [9] applied the theory of planned behavior to understand the PEB of tourists visiting a nature education area in Nantou, Taiwan. 84% of the variances of PEB were explained by the model with attitude having the strongest influence, followed by subjective norms and perceived behavioral control. Situmorang et al. [10] incorporated the new environmental paradigm scales into the theory of reasoned action to understand the mitigation behavior on climate change of residents from Taichung, Taiwan. Results showed that subjective norms greatly influenced behavioral intentions, accounting for 73% of the variances while behavioral intentions moderately influenced PEB, accounting for 48% of the variances. Tien and Huang [11] examined the attitude and pro-environmental behavioral intention of Taiwanese by analyzing data from the Taiwan social change survey, where women were found to have stronger environmental values and pro-environmental behavioral intention compared to men. 

From the various works regarding the PEB of Taiwanese, the theory of planned behaviors was widely used. The surveyed respondents were mostly specified targeted group of people by geographical location [4,10] or specified roles [7,8,9], with a wide range of accounted variances regarding PEB. In addition, subjective norms, the perceived social pressure to engage in a particular behavior, were noted by several researchers to significantly influenced PEB intentions [6,10]. Taiwan, like most East Asia countries and regions, had a higher degree of collectivism and Confucian influence [12], where values like social harmony and strong family ties as well as philosophical beliefs of harmony with the natural world, could also have significant influences on Taiwanese PEB [13]. Hence, these cultural values and beliefs prevalent in Taiwan might suggest that the VBN theory could provide a more comprehensive framework for understanding PEB in the Taiwanese context.

### 1.2. VBN Theory and the Inclusion of Social Norms

Based on decision-making theory, the VBN theory constructed a theoretical model that influenced PEB [14,15]. The model focused on “values” orientation (biospheric, egoistic, altruistic and openness to change values) as well as “beliefs” regarding an ecological (new environmental) world view, awareness of consequences and ascription of responsibility. This in turn led to personal “norms” involving a sense of obligation to engage in pro-environmental actions and finally pro-environmental behavioral actions (including activism, non-activist public-sphere behaviors, private-sphere behaviors as well as behaviors in organizations). The VBN theory has been extensively applied in the environmental field, covering topics involving implementation of sustainable car usage [16,17], acceptance of eco-friendly food [18,19,20], conservation of energy usage [21,22] and the protection of natural habitats [23]. 

According to Schwartz [24], personal values refer to “a guiding principle for any behavior based on desirable trans-situational goals, which varied by relative importance” In the studies of PEB, altruistic values (concern for the welfare of others), biospheric values (concern for the environment and ecological welfare), egoistic values (concerned with their own well-being) form the personal value bases and are often applied by researchers [25,26,27]. Openness to change (willingness to pursue new experiences) has been less researched [18,28]. 

A key aspect of the VBN theory involves the understanding that connection between values and environmentalism are influenced by beliefs, which include “folk” ecological beliefs (ecological world view) regarding the entities impacted by environmental conditions (awareness of consequences) and the effectiveness of individual actions in mitigating these threats (ascription of responsibility) [15]. Research [22,29] had shown that personal values better predicted norms and behaviors compared to ecological world view, as personal values reflected a greater range of motivations while ecological world view concentrated on environmental concerns. Several studies exclude the ecological world view from the VBN model, giving a tighter model in explaining pro-behavior behaviors [21,28,30,31]. Similarly, some studies in the literature [18,32,33,34] exclude the ascription of responsibility from the VBN model. 

Personal norms refer to an individual’s internal standards and moral principles toward behaving pro-environmentally [15] and are repeatedly shown to be a significant predictor of PEB [26,28,31,32]. Finally, PEB are actions that could protect the environment from the negative effects of human activities [15]. Among the four PEBs (activism, non-activist public-sphere behaviors, private-sphere behaviors and behaviors in organizations, some research has concentrated on all four behaviors [28], a particular behavior [32,35] or behaviors in general [26,27,33].

For many years, integrating other psychological or sociological constructs into the VBN theory continued to be an emerging field of research, including the addition of social norms [28,36,37,38], psychological empowerment [39], environmental sensitivity [33], knowledge barrier [40] and overall image [41]. Social norms had been identified as an important factor in understanding PEB based on environmental psychology [42]. Social norms described social pressures felt from important others or the general public at large to engage in certain behaviors [28]. People motivated by social norms tend to listen to recommendations from family, friends, co-workers, or contemporaries [8]. There is research showing that an individual’s social norms are derived from their environmental beliefs, which in turn leads to a sense of personal obligation to behave pro-environmentally; in these studies [36,37,38], personal norms served as a mediating variable between social norms and PEB. On the other hand, other researchers [28,43,44] found that when an individual’s personal interests were in line with a collective society, personal norms had a positive influence on social norms, while social norms had a positive influence on PEB; social norms in this case, served as a mediating variable between personal norms and PEB. 

In summary, applying the VBN theory on Taiwanese in order to understand their PEB is an appropriate model, as Taiwanese society emphasizes cultural values and beliefs. However, limited studies in the literature [5] that applied the VBN theory on general Taiwanese were performed more than a decade ago and did not incorporate the highly collective feature of Taiwanese communities [45,46]. The emphasis of this study is to formulate a more parsimonious model of the VBN theory by streamlining some existing variables and integrating social norms in order to establish the constructs shaping the PEB of contemporary Taiwanese. Establishing this behavioral model could be useful for Taiwan’s goal to achieve net-zero carbon emissions by 2050 and could serve as a future reference for neighboring countries with similar collectivism features, such as Vietnam or Cambodia, that are following a similar path of economic and social development. 

## 2. Materials and Methods

### 2.1. Study Design 

**Figure 1:**
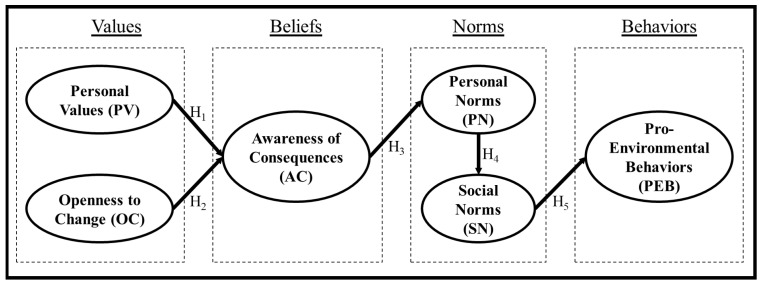
Formulated VBN model.

The formulated VBN model for understanding the PEB of contemporary Taiwanese is shown in Figure 1. Personal values formed the basis of the “value” orientation while openness to change was included to understand its influences due to a relatively low inclusion in various studies in the literature [18,28]. Following the published literature, the ecological world view [21,28,30,31] and ascription of responsibility [18,32,33,34] were excluded to give a more parsimonious “beliefs” orientation. Both personal norms and social norms were included within the “norms” orientation. As Taiwan is a highly collective community, social norms were included as a mediating variable between personal norms and PEB, accordingly to relevant studies in the literature [28,43,44]. Lastly, the general PEB were adopted [26,27,33] to form the “behaviors” variable. 

According to the formulated VBN model, the following hypotheses were proposed:

**Hypothesis 1 (H1):**  *Personal values have a positive influence on awareness of consequences.*

**Hypothesis 2 (H2):**  *Openness to change has a positive influence on awareness of consequences.*

**Hypothesis 3 (H3):**  *Awareness of consequences has a positive influence on personal norms.*

**Hypothesis 4 (H4):**  *Personal norms have a positive influence on social norms.*

**Hypothesis 5 (H5):**  *Social norms have a positive influence on PEB.*

### 2.2. Setting

A simple questionnaire was arranged, and information was collected to test the hypotheses. The questionnaire was surveyed using the proportional stratified random sampling method from the 22 counties, cities, or special municipalities of Taiwan, selecting residential telephone samples from the local telecom’s residential users from October to November 2020, during the evening between 18:30 p.m. and 22:00 p.m. 

### 2.3. Participants 

The survey target population was residents above 18 years old currently living in Taiwan; as of Year 2020, the Taiwan population was estimated to be around 23.6 million, of which 19.9 million were above 18 years old. 

### 2.4. Variables 

The questionnaires used comprised two segments; the first segment had four questions, collecting basic demographic data such as location, age, education level and gender. The second segment had nineteen questions concerning values, beliefs, norms, and behaviors, as shown in Table 1. The four statements on values were basic personal values based on Schwartz (1992) studies. Relationships with the environment were not particularly highlighted. Similarly, the three statements regarding openness to change were general statements and not particularly related to the environment. After careful consideration, more questions were dedicated to personal values, openness to change, and PEB, to strengthen the reliability of internal consistency, as these constructs were believed to encompass more diverse views among Taiwanese.

### 2.5. Measurement 

Each construct contained two to six questions, where all questions were rated using the five-point Likert scale; 1 referred to “strongly disagree” while 5 referred to “strongly agree” according to that particular question. Measures of constructs were modified or created based on previous studies [5,14,15,28,36,37] and discussions with pro-environmental professionals.

### 2.6. Bias

The aim of the questionnaire was explained with clear and concise instructions for respondents, including definitions of terms used in the questionnaire to avoid misunderstandings and misinterpretations. Respondents were ensured that their response would remain anonymous and confidential to encourage honest and accurate replies. Interactions were conducted in Chinese where neutral and balanced language was used to avoid leading or loaded questions that might influence respondents’ responses. Verbal consent was obtained before proceeding with the survey.

### 2.7. Study Size 

According to equation 1 [47], at least 385 completed answered forms were required in order to yield a 5.0% margin of error,

(1) $$n=\frac{z^{2}p\left(1-p\right)N}{e^{2}N+z^{2}p(1-p)}$$

with *n* as the sample size, *z* as the *z*-score concurring to 95% confidence level, *e* as the margin of error and N as the population size. *p*, set as 0.5, was the population associated with the questionnaire. In total, 1079 completed answered forms were collected (as shown in Table 2), greatly exceeding the required minimum. 

### 2.8. Quantitative Variables

Respondents answered 19 questions concerning values, beliefs, norms and behaviors according to the five-point Likert scale. To ensure data quality, the inspection was conducted and repeated, or invalid replies were removed. Descriptive statistical analysis and structural equation modelling (SEM) were performed in order to analyze the data.

### 2.9. Statistical Methods 

SEM served as a statistical technique aimed at determining causal links between constructs, facilitating a straightforward representation of how social psychological factors impact Taiwanese PEB. 

The analysis was conducted using SPSS 25.0 and Amos 20.0 software, where reliability of internal consistency and construct validity were inspected. For the reliability of internal consistency to be at least adequate, the Cronbach’s alpha for the constructs needed to be above 0.70 [48]. However, it is worth noting that the number of items for most of the constructs was less than five, which would violate the tau-equivalence and give a lower reliability coefficient. Hence, the mean inter-item correlation needed to be taken into account, where values should be between 0.150 and 0.500 [49] for constructs, with Cronbach’s alpha lower than 0.70. 

Subsequently, an adequate measurement model of construct validity was developed using the confirmatory factor analysis (CFA) model. The bootstrap approach was further applied. The maximum likelihood method was employed for estimating the models, while covariances were established between exogenous and control variables [48]. Commonly used fit indices were used to measure the overall model fit of the CFA model and formulated VBN model: standardized root mean square residual (SRMR), root mean square error of approximation (RMSEA), Tucker and Lewis index (TLI), and comparative fit index (CFI). Lastly, an examination of the influences of basic demographics was performed using multi-group analysis. 

## 3. Results 

### 3.1. Statistical Fitness

Connections among various constructs could not be directly shown between questions and needed to pass statistical analysis and rational discussion of the variables. Table 3 presents the mean, standard deviation, skewness, and kurtosis of all items. The values of skewness were found to be between −3.0 and +3.0, whereas kurtosis ranged between −10.0 and +10.0, signifying normality [48]. From Table 4, Cronbach’s alpha for the constructs of personal norms and PEB were above 0.70. Although Cronbach’s alpha for the rest of the constructs was below 0.70, their corresponding mean inter-item correlations were between 0.150 and 0.500. Therefore, adequate reliability of internal consistency was obtained. 

Every construct was properly suited for structural regression models within the CFA model, with each having a minimum of two measures. Each item was significantly correlated to its designated construct (*p* < 0.001), exhibiting standardized regression weights beyond 0.4 [48]. The CFA model had values of fit statistics as follows: RMSEA = 0.050 (<0.080); GFI = 0.950 (>0.090); TLI = 0.915 (>0.090); CFI = 0.992 (>0.090), where all values were within the preferred limits [50]. Therefore, adequate construct validity was obtained.

### 3.2. Path Analysis and Standardized Estimates

The SEM for the formulated VBN model had a generally good fit, with RMSEA = 0.076 (<0.080); GFI = 0.908 (>0.090); TLI = 0.806 (>0.090); CFI = 0.833 (>0.090). Except for TLI and CFI, which were almost within the desirable ranges but still satisfactory [51,52], these fit tests were within the appropriate values [50]. The formulated VBN model’s standardized estimates showed that both personal values (*β* = 0.786, *p* < 0.001) and openness to change (*β* = 0.421, *p* < 0.001) had a significantly positive influence toward awareness of consequences. Furthermore, awareness of consequences (*β* = 0.922, *p* < 0.001) had a significant and positive influence on personal norms. Personal norms (*β* = 0.956, *p* < 0.001) had a significant and positive influence on social norms, while social norms (*β* = 0.862, *p* < 0.001) had a significant and positive influence on PEB (as shown in Table 5). The formulated VBN model explained 79.6% of the variance in awareness of consequences, 85.0% of the variance in personal norms, 91.3% of the variance in social norms, and 74.3% of the variance in PEB. 

## 4. Discussion

### 4.1. Formulated VBN Model and Taiwanese PEB

The formulated VBN model’s standard estimates and coefficients of determination validated the VBN theory’s effectiveness in predicting Taiwanese PEB. Moreover, each construct in the causal chain of the formulated VBN model served its role as the mediator between its distal variable and outcome variable. Excluding ecological worldview and ascription of responsibility led to a more parsimonious “beliefs” orientation in the VBN model, resulting in a tighter fit that better predicts Taiwanese PEB. This improvement was evidenced by the high coefficients of determination, exceeding 75% for awareness of consequences (79.6%) and personal norms (85.0%), indicating a substantial level of predictive accuracy [53]. 

In 2011, Chen [5] was the first to confirm that the VBN theory and model were applicable in Taiwan, with results consistent with previous studies by Stern et al. [14] and Stern [15], which were based in Western countries. The coefficients of determination for ecological worldview, awareness of consequences, ascription of responsibility, personal norms and PEB were 63%, 72%, 67%, 72%, and 31%, respectively. A comparison with the tighter formulated VBN model showed higher coefficients of determination, indicating a greater level of predictive accuracy. However, it would be inappropriate to conclude that the formulated VBN model was superior to the original VBN model. Instead, it would suggest that Taiwanese perceptions have evolved over time and the formulated VBN model provided a more accurate prediction of contemporary Taiwanese PEB. 

Results from the formulated VBN model demonstrated that both personal values and openness to change positively influenced awareness of consequences, consistent with the important role that values played in behavior theories [54]. Regarding the role of openness to change, personal values had a stronger influence on awareness of consequences compared to openness to change. This could be due to the strong emphasis by government agencies, civil organizations, community, and family on the collective values of caring for the disadvantages. In Taiwanese society, characterized by a strong sense of collectivism and Confucian influence [12], there was a tendency towards conservatism and a preference for not leading in the adoption of new ideas regarding social innovation. This cultural backdrop might result in a diminished influence of openness to change when compared to personal values, which are deeply rooted in the society’s collective and traditional values. In a similar study, Whitley et al. [18] examined the PEB of undergraduates from Michigan State University in the United States, with respect to biospheric values, altruistic values, egoistic values, tradition values and openness to change influencing beliefs. Among the models of various PEB, the loadings of openness to change were similar to the various personal values in the area of support for pro-environmental policy, energy conservation, food and transportation, but stronger than the various values in the area of recycling. This might be due to the individualistic nature of American society. Ghazali et al. [28] analyzed the PEB of Malay and Chinese residents from Klang valley, Malaysia, with respect to biospheric values, altruistic values, egoistic values, and openness to change. Results showed that the loadings of openness to change were weaker compared to personal values for all residents and solely among Malay residents, but comparable to those of Chinese residents. As the role of openness to change had produced inconsistent results with respect to the different nature (conservative or liberal) of the community [55,56], further research might be needed before more conclusive findings can be determined.

Personal norms refer to an individual’s internal standards and moral principles toward behaving pro-environmentally [15]. Awareness of consequences influence the formation and evolution of personal norms, as individuals might adjust their personal norms accordingly when they become aware of the potential outcomes of their actions. As the Taiwanese community were generally well aware of the negative consequence of not practicing environmental protection (mean of 4.15 out of 5 as seen in Table 4), the results showed that being aware of the negative consequences of not behaving pro-environmentally was sufficient to influence individuals’ personal norms without the mediating role of ascription of responsibility. This direct effect was also observed by Fornara et al. [37], while examining the PEB of residents from South Sardinia, Italy regarding renewable energy sources, where a weak and non-significant linkage between awareness of consequences and ascription of responsibility was found. In a similar manner, Fornara et al. [57] examined the action toward biodiversity and nature conservation of residents from seven European Union countries, where results from their VBN model showed that the direct influence of awareness of consequences on moral norms was stronger than the indirect influence mediated by ascription of responsibility. 

### 4.2. Moderating Role of Social Norms

Results from that formulated VBN model showed that personal norms had a strong and positive influence on social norms, while social norms exhibited a strong and positive influence on PEB. Their respective coefficients of determination were 91.3% and 74.3%, indicating a substantial level of predictive accuracy [53]. When individuals in Taiwanese society strongly adhered to a certain personal principle that was aligned and favored upon by the collective community, they would be more likely to act on this principle, integrating it into their behavior. As the Taiwanese community generally held strong views and actions regarding environmental protection (mean of 4.10 out of 5 as seen in Table 4), personal norms were mediated by the positive influence of social norms, while social norms in turn positively influenced PEB. 

Among the various research studies conducted to understand the PEB of Taiwanese, the theory of reasoned action and theory of planned behavior were widely used and their coefficients of determination for PEB ranged widely, from healthcare personnel in Tsai and Tan [7], undergraduates in Tsai and Tan [8], tourists visiting Nantou in Chang et al. [9] to residents from Taichung in Situmorang et al. [10], giving values of 19%, 30%, 84% and 48%, respectively. Given the complexity and variety of pro-environmental behaviors, the VBN theory and the theory of planned behavior had both seen substantial application and development within the literature. These theories are not necessarily in competition but rather offer complementary perspectives on understanding and promoting PEB. Subjective norms reflect how individuals perceive social expectations, while social norms represent the behaviors and attitudes actually practiced and endorsed by the community. Together, subjective norms and social norms illustrate how individual perceptions and collective behaviors interact, influencing each other and contributing to a better understanding of the collective nature of a society. Due to the restrictive measures during the COVID-19 crisis, Tsai and Tan [7] found that subjective norms had no significant influence on healthcare personnel’s PEB intention. Tsai and Tan [8] found that subjective norms had the weakest influence on PEB intention compared to attitude and perceived behavioral control, as undergraduates viewed PEB as personal decisions regardless of social pressure. However, in the context of the broader Taiwanese public, research by Chang et al. [9] and Situmorang et al. [10] found that subjective norms significantly influence PEB intentions, ranking as the second strongest and strongest construct, respectively. This underscores the critical role that perceived social expectations play in motivating PEB intentions among Taiwanese. The mediating role of social norms in the formulated VBN model echo the findings of Chang et al. [9] and Situmorang et al. [10], highlighting the intricate relationship between community standards and individual behaviors. 

Integrating social norms into the VBN model worldwide, Fornara et al. [37] tried to comprehend the PEB of residents from South Sardinia, Italy regarding renewable energy sources, where social norms positively influenced moral norms, attitude, and trust. Results showed that the expectations of people important to residents would change their personal attitude on renewable energy sources, increase their trust regarding this topic and affect their moral norms to behave in a pro-environmental manner, which in turn positively influenced their PEB. Han and Hwang [36] examined the PEB of convention delegates in the United States, while Al Mamun et al. [38] analyzed the workplace PEB of workers from China regarding energy conservation, where social norms positively influenced personal norms and intention, as incorporating social norms reinforced the role of moral responsibility in prompting their PEB intention. Ghazali et al. [28] considered the PEB of Malaysians from the region of Klang valley and determined that personal norms positively influenced social norms, which in turn positively influenced PEB. The current research supports the findings of Ghazali et al. [28], verifying that, in a collective society that supports environmentalism, social norms serve as the mediating variable between personal norms and PEB. 

### 4.3. Moderating Role of Basic Demographics

The moderating roles of basic demographics were commonly examined in the VBN-related literature, ranging from ethnic groups [28] to product involvement [20] and gender differences [58]. From Table 6, results from the multi-group analysis revealed no significant differences between respondents from the metropolitan and non-metropolitan regions of Taiwan as well as among different levels of education. At the same time, substantial differences were observed among young and mature individuals with regards to personal values influencing the awareness of consequences and awareness of consequences influencing personal norms. Similarly, significant differences were observed between genders with regard to awareness of consequences and personal norms.

The metropolitan and non-metropolitan regions of Taiwan were classified according to the common consensus, with northern Taiwan as the most urbanized region. However, it is important to note that both urban and rural areas exist within all four regions of Taiwan. With Taiwan having 38 urban and 146 rural townships [59], categorizing areas as metropolitan presented challenges due to the diverse and widespread nature of these townships across Taiwan. This diversity complicated efforts to uniformly define metropolitan regions, reflecting the varied urban and rural landscapes within Taiwan. Hence, the non-significant differences between the metropolitan and non-metropolitan regions could be due to the challenges faced in the correct definition of the metropolitan regions of Taiwan. 

Although the Environmental Education Act was only legislated in year 2010, marking the start of formal environmental education in schools, the Environmental Protection Administration was already established in year 1971 and actively promoted environmental protection within the various levels of Taiwanese society. Hence, the exposure of the topic of environmental protection should be similar among young and mature individuals. Studies conducted by Roberts et al. [60] showed that personality characteristics and values changed considerably as individuals aged and matured. Young individuals might still be absorbing new ideas and knowledge, where their personal values were yet to be securely formed. This might account for the significant differences observed between young and mature individuals with regards to personal values and awareness of consequences. However, the literature reported that age was not substantially related to personal values [61]. At the same time, when individuals were more conscious of the environmental effect of their behaviors, they were more likely to develop strong personal norms for the environment. Young individuals might be more likely to strengthen their personal norms upon gaining an understanding of their consequences compared to the mature individuals, since they might need to experience the adverse effects of unsustainable behaviors for a much longer duration of time.

Similarly, the strong informal environmental education in Taiwan supplemented for the late implementation of formal environmental education, accounting for the non-significant differences between low and high education levels among the respondents, which was similar to that seen in the published literature [62,63].

According to Dietz et al. [64], men typically had a greater, self-centered mindset and focused on the personal advantages that PEB could provide. Women placed a stronger emphasis on altruism and the effects of PEB on the community. Similarly, as reported by Tien and Huang [11] regarding gender differences in Taiwan, men showed a greater willingness to support environmental protection through higher taxes, while women were more inclined to adjust their lifestyle, even if it meant a lower standard of living, for the sake of environmental conservation. This self-centered mindset, prevalent in Taiwan’s gender differences, might explain the significant differences observed between male and female respondents with regards to awareness of consequences and personal norms, which was also reported in Yang et al. [58] and Zhao et al. [65]. As a result, men might have a greater perception of obligation to undertake PEB based on their awareness of the corresponding consequences and impacts on their personal future. Women, on the other hand, might emphasize the awareness of the corresponding consequences and impacts on their collective community.

## 5. Conclusions

This study utilized pertinent theoretical frameworks and empirical findings, subjecting them to data analysis verification. The implementation of the tighter formulated VBN theory and model provided a more comprehensive understanding of the social–psychological factors influencing the PEB of contemporary Taiwanese. With respect to Hypothesis 1 and Hypothesis 2, personal values and openness to change both positively influenced awareness of consequences, with personal values having a stronger loading. In addition, Hypothesis 3, Hypothesis 4, and Hypothesis 5 were verified, where awareness of consequences positively influenced personal norms, which in turn positively influenced social norms, with social norms positively influencing PEB. 

With regards to the mediating roles of the basic demographics, the findings indicated that location, age, education level, and gender were generally statistically limited, but differed in three relationships: mature individuals had a stronger tendency towards awareness of consequences due to personal values, the young had a stronger tendency towards personal norms due to awareness of consequences, and men had a stronger tendency towards personal norms due to awareness of consequences, while women had a weaker tendency due to a greater emphasis on altruism. 

Pro-environmental interventions and programs in Taiwan could be examined, specifically when corresponding to proven behavior and relevant theories of behavioral change. Future intervention approaches, such as sharing of personal, pro-environmental lifestyle through word of mouth or social media, periodically decluttering personal items and keeping a minimalist lifestyle, where these personal norms are in line with collective social norms, could help to strengthen PEB. Furthermore, with respect to the mediating roles of the basic demographics, future possible advances, such as the shaping of individual’s personal values at a younger age, could develop and advance PEB by refining the associated social–psychological indications. Furthermore, emphasizing the consequences of environmental degradation and their negative impacts on an individual’s personal lifestyle according to genders could be useful; for example, by informing men regarding the consequences of increasing personal expenses due to wastage, adverse effects on personal health due to pollution, decrease in personal quality of life due to a degraded environment as well as by informing women regarding increasing family expenses due to wastage, adverse effects on family members’ and neighbors; health due to pollution, and a decrease in community cleanness due to a degraded environment. These deductions might produce a better comprehension of the environmental behaviors of contemporary Taiwanese and hence play a part in promoting PEB.

Finally, this study identified several limitations, including reliance on subjective self-reported questionnaires and not observing PEB in real-life scenarios. The VBN theory, focusing on cognitive processes, might overlook emotional, unconscious, and situational influences on PEB, which was diverse in nature and varied in difficulty, visibility, and personal cost. Furthermore, the VBN theory did not account for how its constructs evolved over time or reacted to new information. Therefore, the formulated VBN model’s findings should be considered within the context of contemporary Taiwanese or countries with similar socio-economic settings.

## Figures and Tables

**Table 1:** List of constructs and items.

Item	Statement
	Personal values
PV1	I hope that my inner mind is rich and contented.
PV2	I hope that I have a better capability to induce changes, compared to my current abilities
PV3	In this world, all living beings have the equal rights to survive.
PV4	I believe that human beings need to live in harmony with other beings in the natural world.
	Openness to Change
OC1	We should respect the values of diverse views and not discriminate against or criticize others with different opinions.
OC2	In general, I believe that society must continue to reform in order to progress; even if reform is risky, I will still support reform.
OC3	Taiwan’s environmental policies should be in line with the policy trends of other countries.
	Awareness of Consequences
AC1	I believe that our Earth’s resources such as oil, coal mines and natural gas will be used up eventually.
AC2	I believe that global warming is caused by humans.
	Personal Norms
PN1	I believe that I should conserve electricity.
PN2	I believe that I will have a bad conscience, unless I always take into consideration the living environment and resource needs of the next generation.
	Social Norms
SN1	My family and friends around me are working hard to protect the environment.
SN2	Many well-known and respected people in our society are advocating energy conservation and reducing carbon emissions.
	Pro Environmental Behaviors (PEB)
PEB1	I have planned or actually participated in environmental protection activities, such as community recycling or beach cleanup.
PEB2	I will purchase products that do not contain environmentally damaging components (for instance chlorofluorocarbons).
PEB3	I will keep my daily necessities as simple as possible in order to avoid unnecessary waste.
PEB4	I will choose to use recycled items, such as recycled paper, second-hand clothes.
PEB5	I will travel using the mass public transportation system.
PEB6	I will try to reuse items in order to reduce waste.

**Table 2:** Basic demographics of sample.

Variables		*n* = 1079	Percentage
*Location*			
Metropolitan	Northern Taiwan	484	44.9%
Non-Metropolitan	Central Taiwan	263	24.4%
	Southern Taiwan	292	27.1%
	Eastern Taiwan	25	2.3%
	Outlying Islands	15	1.4%
*Age*			
Young	18–29	88	8.2%
	30–39	114	10.6%
Mature	40–49	258	23.9%
	50–59	253	23.4%
	60–69	227	21.0%
	>70	138	12.8%
Declined to disclose		1	0.1%
*Education level*			
Low	Elementary and below	115	10.7%
	Middle school	104	9.6%
	High school	305	28.3%
High	Vocational training	174	16.1%
	Bachelor’s degree	304	28.2%
	Masters and Ph.D.	73	6.8%
Declined to disclose		4	0.4%
*Gender*			
Male		480	44.5%
Female		599	55.5%

**Table 3:** Statistical analysis of items.

Constructs	Item	Mean	S.D.	Skewness	Kurtosis	*β*
Personal values	PV1	4.00	0.802	−1.347	2.367	0.549
	PV2	3.88	0.829	−1.363	1.928	0.523 ***
	PV3	4.01	0.875	−1.299	1.719	0.602 ***
	PV4	4.22	0.659	−1.227	3.797	0.695 ***
Openness to Change	OC1	4.16	0.709	−1.352	3.507	0.505
	OC2	3.88	0.874	−1.197	1.343	0.651 ***
	OC3	3.88	0.900	−1.306	1.596	0.635 ***
Awareness of Consequences	AC1	4.10	0.965	−1.323	1.443	0.394
	AC2	4.20	0.842	−1.446	2.485	0.483 ***
Personal Norms	PN1	4.40	0.607	−1.308	5.083	0.688
	PN2	4.17	0.779	−1.539	3.749	0.651 ***
Social Norms	SN1	3.94	0.837	−1.427	2.263	0.410
	SN2	3.48	1.118	−0.658	−0.766	0.267 ***
Pro-Environmental Behaviors	PEB1	3.99	0.848	−1.499	2.737	0.454
	PEB2	4.12	0.733	−1.464	3.822	0.605 ***
	PEB3	4.32	0.554	−0.767	4.322	0.724 ***
	PEB4	3.90	0.868	−1.311	1.646	0.476 ***
	PEB5	4.05	0.794	−1.326	2.311	0.506 ***
	PEB6	4.23	0.613	−1.172	4.827	0.707 ***

Note: S.D. denoted standard deviation and *β* indicated standardized regression coefficients for the formulated VBN model. *** *p* < 0.001.

**Table 4:** Statistical analysis of constructs.

Constructs	Mean	S.D.	Cronbach’s Alpha Coefficient	Mean Inter-Item Correlation
Personal values	4.03	0.804	0.681	0.348
Openness to Change	3.97	0.843	0.620	0.352
Awareness of Consequences	4.15	0.907	0.558	0.387
Personal Norms	4.29	0.708	0.708	0.548
Social Norms	3.71	1.012	0.491	0.325
Pro-Environmental Behaviors	4.10	0.757	0.768	0.355

Note: S.D. denoted standard deviation.

**Table 5:** Path analysis of constructs.

Hypothesis	Paths	Estimate	*p*-Value	R^2^
H1	AC ← PV	0.786	<0.001 ***	
H2	AC ← OC	0.421	<0.001 ***	79.6%
H3	PN ← AC	0.922	<0.001 ***	85.0%
H4	SN ← PN	0.956	<0.001 ***	91.3%
H5	PEB ← SN	0.862	<0.001 ***	74.3%

Note: R^2^ denoted coefficients of determination. *** *p* < 0.001.

**Table 6:** Multi-group analysis for various basic demographics.

Hypothesis	Path Coefficient			χ^2^/Df	Results
	Metropolitan	Non-Metropolitan	Differences		
AC ← PV	0.800 ***	0.759 ***	0.041	0.156	Metropolitan = Non-Metropolitan
AC ← OC	0.374 ***	0.473 ***	−0.099	0.786	Metropolitan = Non Metropolitan
PN ← AC	0.907 ***	0.928 ***	−0.021	0.440	Metropolitan = Non-Metropolitan
SN ← PN	0.952 ***	0.957 ***	−0.005	0.022	Metropolitan = Non-Metropolitan
PEB ← SN	0.822 ***	0.904 ***	−0.082	0.498	Metropolitan = Non-Metropolitan
**Hypothesis**	**Path Coefficient**			**χ^2^/Df**	**Results**
	**Young**	**Mature**	**Differences**		
AC ← PV	0.769 ***	0.772 ***	−0.003	4.515 *	Young < Mature
AC ← OC	0.407 **	0.428 ***	−0.021	1.816	Young = Mature
PN ← AC	0.982 ***	0.926 ***	0.056	7.096 **	Young > Mature
SN ← PN	0.936 ***	0.973 ***	−0.037	0.002	Young = Mature
PEB ← SN	0.930 ***	0.845 ***	0.085	0.816	Young = Mature
**Hypothesis**	**Path Coefficient**			**χ^2^/Df**	**Results**
	**Low Edu**	**High Edu**	**Differences**		
AC ← PV	0.655 ***	0.836 ***	−0.181	0.083	Low = High
AC ← OC	0.518 ***	0.359 ***	0.159	1.746	Low = High
PN ← AC	0.938 ***	0.931 ***	0.007	2.327	Low = High
SN ← PN	0.941 ***	0.969 ***	−0.028	0.147	Low = High
PEB ← SN	0.929 ***	0.822 ***	0.107	3.007	Low = High
**Hypothesis**	**Path Coefficient**			**χ^2^/Df**	**Results**
	**Male**	**Female**	**Differences**		
AC ← PV	0.835 ***	0.722 ***	0.113	0.014	Male = Female
AC ← OC	0.399 ***	0.437 ***	−0.038	0.091	Male = Female
PN ← AC	0.953 ***	0.914 ***	0.039	8.581 **	Male > Female
SN ← PN	0.975 ***	0.922 ***	0.053	3.765	Male = Female
PEB ← SN	0.828 ***	0.908 ***	−0.080	0.982	Male = Female

Note: * *p* < 0.05, ** *p* < 0.01, *** *p* < 0.001.

## Data Availability

The data presented in this study are available on reasonable request from the corresponding author. The data are not publicly available due to privacy issues.
